# Clinical performance of alkasite and glass-hybrid restorations compared with resin composite in class II cavities: a randomized clinical trial

**DOI:** 10.1007/s00784-026-06869-w

**Published:** 2026-05-02

**Authors:** Kemal Işıklı, Uzay Koc Vural, Filiz Yalcin Cakir

**Affiliations:** Department of Restorative Dentistry, School of Dentistry, Hacettepe University, Altındağ, Ankara, 06230 Türkiye

**Keywords:** Alkasite, Glass-hybrid, Composite resin, Clinical performance, FDI criteria

## Abstract

**Objectives:**

The aim of this randomized clinical trial was to evaluate the 18-month clinical performance of alkasite and glass-hybrid restorations compared with resin composite in Class II restorations.

**Materials and methods:**

A total of 50 patients requiring at least three Class II restorations in premolar and molar teeth were recruited. Each patient received three restorations, which were randomly assigned to one of the following materials: an alkasite (Cention N, Ivoclar Vivadent), a glass-hybrid (Equia Forte HT, GC Corp.), or a resin composite (Gradia Direct Posterior, GC Corp.). Alkasite and glass-hybrid served as test groups while resin composite served as the control group. During the 18-month follow-up, restorations were scored at baseline, 6, 12, and 18 months using the FDI criteria. Data were analyzed using the Chi-square and Cochran’s Q tests (α = 0.05).

**Results:**

No significant differences were detected among the groups for esthetic, functional, or biological criteria over 18 months (*p* > 0.05). Regarding esthetic properties, the control group showed 100% success for all esthetic criteria at all recall visits, while the alkasite group maintained a 96% success rate at all time points, and glass-hybrid showed 98% success at 6 and 12 months and 92% at 18 months. Minor score-2 changes in color match and gloss were detected in the glass-hybrid and alkasite groups but were not significant (*p* > 0.05). For functional and biological outcomes, all groups achieved 100% success rates for all evaluated parameters.

**Conclusions:**

Alkasite and glass-hybrid groups exhibited clinical performance comparable to resin composite over the 18-month follow-up in Class II cavities. All materials demonstrated excellent functional and biological stability while achieving clinically acceptable aesthetic results.

**Supplementary Information:**

The online version contains supplementary material available at 10.1007/s00784-026-06869-w.

## Introduction

 Restorative dentistry is a continuously evolving field driven by advances in biomaterial science [1, 2]. The main goal is to fix the shape, function, and color of teeth damaged by caries or structural disorders while preserving as much healthy dental tissue as possible [3].

Numerous extensive systematic reviews and meta-analyses validate that the standard annual failure rate for direct posterior resin composite restorations ranges from 1% to 3%. This range is evident in both short-medium term (1–5 years) and long-term (up to 20 + years) studies, remaining stable irrespective of the composite material used, as long as modern techniques and materials are applied [4]. Therefore, particularly over the last few decades, restorative ideas have changed from just putting mechanical parts in place of damaged tissue to methods that are less invasive and more integrated with biology [5]. This shift has driven the search for materials that not only replace lost tissues but also interact with dental structures through biological and chemical processes, giving rise to bioactive restorative materials [6]. In dentistry, bioactivity is the ability of a material to cause certain biological responses, such as the formation of an apatite-like layer, ion exchange, or antibacterial effects [7, 8]. Bioactive materials can neutralize acids, keep the pH level stable, help remineralize dentin that has lost minerals, and make strong connections with mineralized tissues in the mouth [9]. These features are especially important for restorations in posterior stress-bearing areas, which are always under mechanical stress and acidic challenges [10, 11].

In the field of minimally invasive dentistry, alkasite and glass-hybrid restorative materials represent a step forward as modern bioactive restorative systems [6, 12]. New glass ionomer technology is used to make glass-hybrid materials. This technology uses glass particles that are highly reactive. They release fluoride over time and chemically bond to teeth. They are considered suitable for patients at high risk of cavities. Clinical and laboratory studies indicate that glass-hybrid systems, which are essentially reinforced high‑viscosity glass ionomer cements, provide higher compressive strength, improved wear resistance, and superior marginal integrity compared with conventional glass ionomer cements [12–14]. These properties make them suitable candidates for posterior restorations in high caries‑risk patients. However, despite these favorable in vitro and short‑term clinical outcomes, robust evidence regarding their long‑term clinical durability in Class II restorations under significant occlusal load remains limited [15, 16].

In contrast, alkasite‑based restorative materials are resin‑based, bulk‑fill systems containing functional alkaline glass fillers dispersed in a dimethacrylate resin matrix [17, 18]. These fillers actively release calcium, hydroxide, and fluoride ions, leading to an increase in local pH, buffering of acidic challenges, and promotion of enamel and dentin remineralization as demonstrated by microhardness testing and SEM analyses [13, 14, 17–20]. Their ion‑release profile allows them to counteract acidic attacks and stabilize the pH at the tooth–restoration interface, thereby helping to inhibit demineralization and support apatite formation [17, 18, 20].

Clinically, both material classes are positioned as bioactive options for posterior restorations, particularly in high caries‑risk or plaque‑retentive areas. Glass‑hybrid systems are mainly indicated as semi‑permanent or medium‑term restorations in stress‑bearing areas, whereas alkasites are marketed as bulk‑fill alternatives to amalgam and resin composite for Class I and II restorations in primary and permanent teeth [17, 18, 21]. Nevertheless, for both glass‑hybrids and alkasites, the novelty and added value of the present study lie in providing additional comparative data on their performance in Class II cavities subjected to occlusal stress, thereby addressing the current gap in long‑term clinical evidence and helping to clarify their indications and limitations relative to conventional restorative materials.

However, the effects of a material observed under laboratory conditions can only be meaningfully interpreted and translated into clinical recommendations when they are supported by well-designed clinical studies. Thus, this randomized controlled clinical trial aimed to evaluate the 18-month clinical performance of an alkasite and a glass-hybrid, in comparison to a micro-filled resin composite in Class II cavities according to the FDI criteria. The null hypothesis tested was that there would be no significant difference in the clinical performance among the three restorative materials after 18 months of clinical service.

## Materials and methods

### Ethical considerations

Ethical approval was granted by the Clinical Research Ethics Committee of Hacettepe University, and all clinical procedures were performed at the Department of Restorative Dentistry, School of Dentistry, Hacettepe University, Ankara, Türkiye (No: 2022/07 − 03, dated 26.04.2022) and Regulation on Medical Device Clinical Trials (Official Gazette No. 29111, dated 06.09.2014). Permission to conduct the study was granted by the Turkish Medicines and Medical Devices Agency (TITCK) (Approval No: E-68869993-511.06-804783, dated 04.07.2022). The study was also registered at clinicaltrials.gov under the number of NCT06211582. Before providing written informed consent, all patients were thoroughly informed about the purpose of the study. The clinical trial was conducted in full accordance with the principles of the Declaration of Helsinki and Good Clinical Practice guidelines. A written consent was obtained from the patients.

### Study design

A three-arm within-subject design was used, with three restorative approaches tested within the same mouth. The study was designed as a prospective, longitudinal investigation (18-month follow-up) with equal within-subject allocation and concealed, randomised assignment to the three treatment groups. Patients were recruited over a 12-month period (August 2022 to August 2023) from patients who attended Restorative Dentistry clinics of University and required three Class II restorations in the premolar and molar region of the same mouth. All patients were initially examined at the Department of Oral Diagnosis and radiographic examinations were obtained at the Department of Oral and Maxillofacial Radiology. The indication for Class II restorations and eligibility for inclusion were subsequently confirmed by the investigators at the Department of Restorative Dentistry. The inclusion and exclusion criteria are summarised in Table 1.

**Table 1:** Inclusion/exclusion criteria

Category	Criteria
Inclusion	•Adults 18–65 years, systemically healthy, permanent dentition • ≥3 vital posterior teeth (molar/premolar) requiring proximal restorations (D₂) • Vital teeth with sufficient remaining sound enamel and dentin to support adhesive restoration • Class II restorations with adjacent/antagonistic contacts and ≥ 1 functional occlusal contact • Patients with functional occlusion and at least 28 natural teeth • Favourable and stable occlusal relationship between the remaining teeth • Ability to attend follow-up visits
Exclusion	**•** Pregnancy or lactation • Unstable systemic disease; drug/alcohol abuse • Advanced/unresolved periodontal disease • Severe bruxism or significant malocclusion • Allergy to resin-based materials/adhesives • Pulp exposure or irreversible pulp pathology • Use of removable partial dentures or opposing full dentures/fixed prostheses

### Sample size calculation

Sample size estimation was performed using a package G*Power version 3.1. A significance level of α = 0.05, a statistical power (1 − β) = 0.95, and an effect size of w = 0.5 were adopted and Chi-square test was run. The analysis indicated that a minimum of 62 restorations would be required. To increase the power of the study and to compensate for possible exclusions or losses, a total of 150 restorations (50 restorations per group) were included.

### Randomization

Random allocation was performed using a computer-generated sequence obtained from Randomizer.org. Each enrolled participant received three restorations, with the placement order determined according to the pre-generated randomization scheme. Allocation concealment was ensured by enclosing the sequence in sealed, opaque envelopes, which were opened only after participant eligibility had been confirmed. No further blinding procedures were implemented following allocation. All participants ultimately received three restorative interventions. Alkasite (Cention N, Ivoclar Vivadent, Schaan, Liechtenstein) and glass-hybrid (Equia Forte HT, GC, Tokyo, Japan) groups served as test and resin composite (Gradia Direct Posterior, GC, Tokyo, Japan) served as control. The characteristics of the materials and the patients’ are shown in Tables 2, 3  and 4.

**Table 2:** Composition, type and manufacturer of the materials tested

Material	Manufacturer	Main Components	Lot No
Equia Forte Fil HT	GC Corp., Tokyo, Japan	Powder: 95% strontium fluoroaluminosilicate glass, 5% polyacrylic acid Liquid: 40% aqueous polyacrylic acid	2405281
Equia Forte Coat	GC Corp., Tokyo, Japan	50% methyl methacrylate, 0.09% camphorquinone	2406121
Cention N	Ivoclar Vivadent, Schaan, Liechtenstein	Calcium fluorosilicate glass, barium-aluminum silicate glass, ytterbium trifluoride, UDMA-based copolymer, Ivocerin initiator system	Z0233Y
Gradia Direct Posterior	GC Corp., Tokyo, Japan	UDMA matrix with dimethacrylates, fluoro-aluminosilicate glass, silica fillers, camphorquinone initiator	1907192
Solare Universal Bond	GC Corp., Tokyo, Japan	10-MDP, 4-META, UDMA, acetone, silanized colloidal silica, water	1904041

*UDMA Urethane dimethacrylate, *10-MDP* 10-Methacryloyloxydecyl dihydrogen phosphate, *4-META* 4-Methacryloxyethyl trimellitate anhydride

**Table 3:** Summary of the clinical application steps for alkasite (Cention N), glass-hybrid (Equia Forte HT), and resin composite (Gradia Direct Posterior), outlining surface conditioning, insertion technique, curing protocol, and final finishing procedures

Alkasite Cention *N* Ivoclar Vivadent, Schaan, Liechtenstein	One scoop powder and 2 drop liquid were dispensed side-by-side on a mixing pad using a plastic spatula for approximately 45 s. The material was applied to the cavity in bulk, and condensed. Self-curing was allowed for 4 min, after which light polymerization was performed for 40 s using an LED curing unit (D-Light, GC corp., Tokyo, Japan) operating at 1200 mW/cm². The output irradiance of the LED curing unit was verified using a radiometer prior to the restorative procedures to ensure consistent light intensity. Occlusion was verified using double-sided articulating paper (blue for occlusion and red for articulation; 20 μm). Premature contacts and occlusal interferences were selectively adjusted using fine-grit diamond finishing burs, followed by polishing with discs and rubber polishers to restore proper occlusal morphology and surface smoothness. Final verification confirmed balanced occlusal contacts and the absence of interferences during functional movements. Cervical adaptation and proximal contact were assessed with dental floss and corrected, when necessary, using flexible polishing discs (952.900.140 and Compo System, Komet). The same occlusal adjustment protocol was applied to all restorative groups.
Glass-Hybrid EQUIA Forte HT, GC Corp., Tokyo, Japan	The cavity was conditioned with 20% polyacrylic acid for 10 s (Cavity Conditioner, GC corp., Tokyo Japan), thoroughly rinsed, and gently air-dried without desiccation. Glass-hybrid (EQUIA Forte HT, GC corp., Tokyo, Japan) capsule was triturated for 10 s in a capsule mixer and the material was inserted in bulk using a capsule applicator. After the initial 2.5-min setting time, occlusion was verified as described above. Then, a uniform layer of coating material (Equia Forte Coat; GC corp., Tokyo, Japan) was applied and light-cured for 20 s with the same LED curing unit to ensure standardization of the polymerization procedure. The final restoration was checked as described above.
Resin composite Gradia Direct Posterior, GC Corp., Tokyo, Japan	The restorative procedure was initiated by selectively etching the enamel margins with 37% orthophosphoric acid gel for 30 s, followed by thorough rinsing and gentle air-drying. Solare Universal Bond (GC corp., Tokyo, Japan) was then applied for 10 s, gently air-thinned for 5 s, and light-cured for 10 s. Resin composite material was placed in increments not exceeding 2 mm. Each increment was light-cured for 20 s with same LED device. The restorations were finished and polished under water cooling using flexible discs (952.900.140 and Compo System, Komet). The final restoration was checked as described above.

**Table 4:** Characteristics of the patients

Patient Age Range*	*n*	%
18–30	48	95,9
30–60	2	4,1
Sex		
Female	26	52.0
Male	24	48.0

*X̄ ± SD = 23.4 ± 5.7, min–max = 18–45

### Interventions

A single trained operator was performed operative procedures and clinical documentation of all restorative procedures. Documentation was achieved through standardized digital photography from direct, occlusal and buccal views (Nikon D5600, Japan). Tooth surfaces were meticulously cleaned to eliminate dental plaque and the salivary pellicle using a fluoride-free prophylaxis paste (Dentsply Sirona, York, Pennsylvania, USA) applied with a polishing brush mounted on a slow-speed handpiece. The patients were given local anesthesia to manage pain, when needed. The isolation was achieved using rubber dam (OptraDam; Ivoclar Vivadent, Schaan, Liechtenstein).

Cavity preparations were performed according to minimally invasive principles and were limited to the extent of the carious lesion as determined clinically and radiographically. Only cavities compatible with the clinical indications of the tested restorative materials were included. Extremely small or excessively large cavities, as well as cavities involving complete cusp coverage, were excluded to maintain comparability among restorations. Cavity preparation was performed under water-cooling using high-speed spherical and cylindrical diamond burs (1.204.023 and 9120.314; Komet, Lemgo, Germany). Remaining carious tissue was removed with hand instruments and/or slow-speed round burs proceeding from the periphery toward the center. The cavity walls were rounded at the internal line angles, no bevels were placed. The missing walls of the cavities restored using a pre-contoured sectional matrix system (Palodent V3, Dentsply Sirona, Charlotte, USA). All materials were placed in the same session according to the manufacturer’s instructions (Table 3). However, after cavity preparation, if any doubt arose regarding compliance with the inclusion criteria, the patient was treated but not included in the study. Cases in which the lesion depth was found to be either deeper or shallower than the predefined inclusion criteria were also excluded.

### Clinical evaluation

Two independent and calibrated examiners who were unaware of the material type conducted the evaluations. Inter-examiner reliability for the recorded evaluations was quantified using Cohen’s kappa statistic to determine the level of agreement between assessors (κ). Prior to the clinical evaluations, the examiners underwent a calibration session during which 20 restorations were jointly assessed according to the FDI criteria. The level of agreement was calculated using Cohen’s kappa statistics, demonstrating excellent intra-examiner (κ = 0.98) and inter-examiner (κ = 0.95) reliability. In instances where the two examiners disagreed, the restoration was jointly re‑evaluated and a final score was established through a consensus process.

Each restoration was rated using a five-point ordinal scale: score 1, excellent clinical performance; score 2, clinically good; score 3, satisfactory; score 4, unsatisfactory but reparable; and score 5, failure necessitating replacement.

The primary outcomes were the survival rate and the success rate of the restorations. The survival was defined as a restoration not requiring replacement (FDI scores of 1–4) and the success as a restoration not needing replacement or repair (FDI scores of 1–3). The secondary outcomes of the trial were the FDI properties of the three materials. Each restoration was systematically recorded through standardized photographic documentation obtained from direct, occlusal and buccal views, both prior to and following treatment, as well as at each scheduled follow-up visit.

### Statistical analysis

SPSS version 22.0 (IBM Corp., Armonk, NY, USA) was run for analyses. Descriptive statistics and frequency distributions were calculated.

Cochran's Q test was used to compare changes within groups across time points (1 week, 6, 12, and 18 months), and Chi-square tests were used to compare differences among groups at each interval. A p value < 0.05 was considered statistically significant.

## Results

**Fig. 1:**
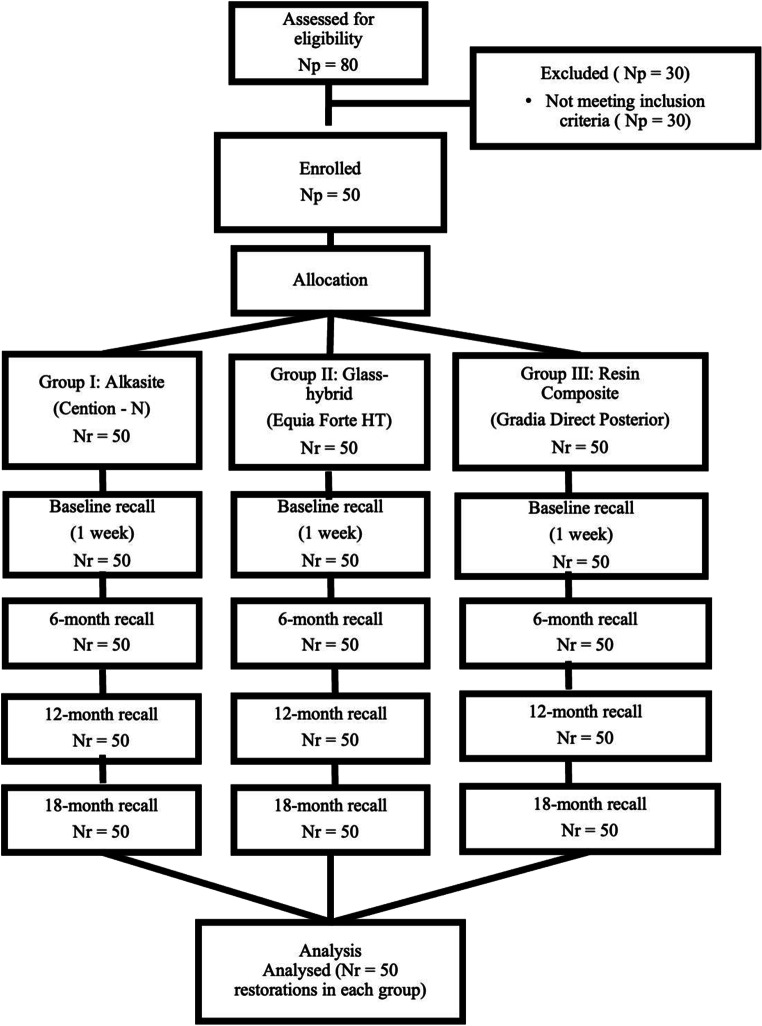
Flow chart. Np: number of patients, Nr: number of restorations.

The progression of the study is illustrated in Fig. 1. Of the 80 individuals initially screened for eligibility, 50 fulfilled the predefined inclusion and exclusion criteria and were subsequently enrolled in the study (Table 1).

Table 4 summarizes the demographic information of the patients. The distribution of restored teeth according to tooth type was as follows: 83 restorations were placed in maxillary premolars, 26 in maxillary molars, 26 in mandibular premolars, and 15 in mandibular molars, yielding a total of 150 restorations. The overall recall rate was 100% at baseline, 6, 12, and 18 months.

### Surface gloss and roughness

**Fig. 2:**
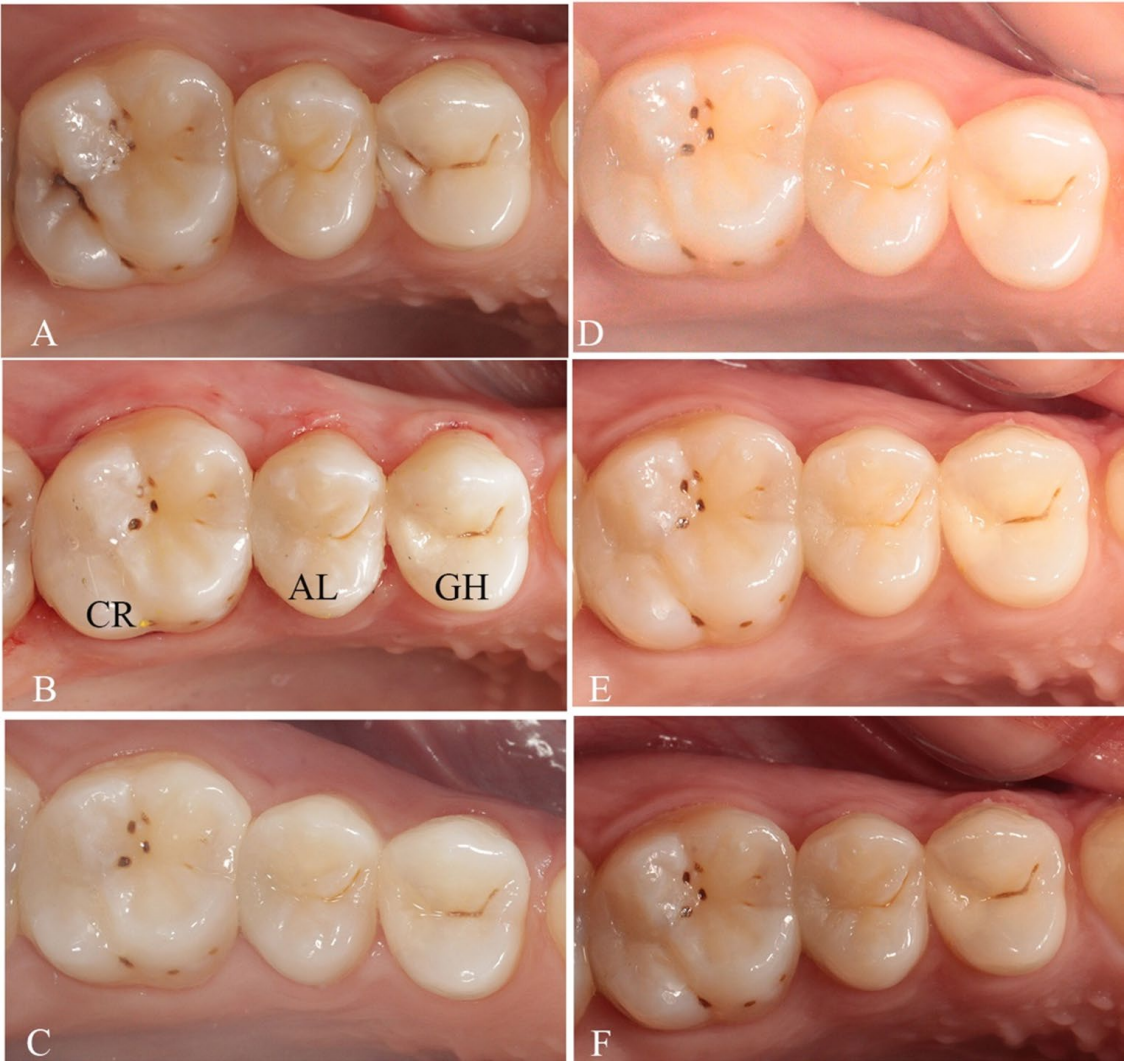
Class II caries lesions in teeth 24, 25, 26 (**A**) Pre-operative, (**B**) Post-operative restorations, (**C**) 1-week, (**D**) 6-month, (**E**) 12-month, and (**F**) 18-month. In this case, glass-hybrid was placed on the distal surface of tooth #24, alkasite on the distal surface of tooth #25, and composite resin restorative material on the distal surface of tooth #26. (AL: Alkasite, CR: Composite Resin, GH: Glass-Hybrid)

The glass-hybrid group showed success rates of 100% at baseline, 98% at 6 and 12 months, and 92% at 18 months (one restoration received an FDI score of 2 at 6 and 12 months and four restorations received a score of 2 at 18 months). The alkasite group demonstrated a 96% success rate at all recall periods (two restorations received a score of 2 at 6, 12, and 18 months). Control group showed a 100% success rate throughout the study. Fisher’s exact test revealed no significant differences at 6, 12, and 18 months in either glass-hybrid or alkasite group compared to control (*p* > 0.05, Fig. 2).

### Color match

At baseline and at the 6-month recall, all restorations showed a 100% success rate in all groups. At 12 months, a 96% success rate was achieved in both the glass-hybrid and alkasite groups (two restorations in each group received a score of 2). At 18 months, the success rate was 90% (five restorations received a score of 2) in the glass-hybrid group, 96% (two restorationsreceived a score of 2) in the alkasite group, and 100% in the control group. Fisher’s exact test showed no statistically significant differences at any recall period among the groups (*p* > 0.05).

### Anatomical form, surface and marginal staining

All restorations in the glass-hybrid, alkasite, and resin composite groups showed a 100% success rate across all evaluations.

### Functional and biological criteria

All restorations in the glass-hybrid, alkasite, and resin composite groups demonstrated a 100% success rate throughout the 18-month follow-up period. No failures were recorded for retention, marginal adaptation, wear resistance, proximal contact, radiographic evaluation or patient satisfaction at baseline, 6, 12, and 18 months evaluations. Similarly, none of the restorations exhibited postoperative sensitivity, secondary caries, adverse periodontal response, or loss of pulp vitality during the observation period. No adverse events or unintended effects were observed during the follow-up period. The distribution of FDI evaluation scores for the three restorative materials across the follow-up periods is summarized in Table 5.

**Table 5:** Summary of FDI esthetic, functional, and biological evaluation scores for glass-hybrid, alkasite, and composite resin restorations at baseline and follow-up recalls, including intergroup comparisons. (Chi-square test)

	Recall	Glass-hybrid (*n*)	Alkasite (*n*)	Resin Composite (*n*)	*p* value
Esthetic Criteria					
Surface gloss and roughness	Baseline	50 (1)	50 (1)	50 (1)	n/a
	6 months	49 (1) / 1 (2)	48 (1) / 2 (2)	50 (1)	0.773
	12 months	49 (1) / 1 (2)	48 (1) / 2 (2)	50 (1)	0.547
	18 months	46 (1) / 4 (2)	48 (1) / 2 (2)	50 (1)	0.773
Color match	Baseline	50 (1)	50 (1)	50 (1)	n/a
	6 months	50 (1)	50 (1)	50 (1)	0.166
	12 months	48 (1) / 2 (2)	48 (1) / 2 (2)	50 (1)	0.070
	18 months	45 (1) / 5 (2)	48 (1) / 2 (2)	50 (1)	0.166
Anatomical form	Baseline / 6 / 12 / 18 months	50 (1)	50 (1)	50 (1)	n/a
Surface and marginal staining	Baseline / 6 / 12 / 18 months	50 (1)	50 (1)	50 (1)	n/a
Functional Criteria					
Retention	Baseline / 6 / 12 / 18 months	50 (1)	50 (1)	50 (1)	n/a
Marginal adaptation	Baseline / 6 / 12 / 18 months	50 (1)	50 (1)	50 (1)	n/a
Wear resistance	Baseline / 6 / 12 / 18 months	50 (1)	50 (1)	50 (1)	n/a
Proximal contact point	Baseline / 6 / 12 / 18 months	50 (1)	50 (1)	50 (1)	n/a
Radiographic evaluation	Baseline / 6 / 12 / 18 months	50 (1)	50 (1)	50 (1)	n/a
Patient satisfaction	Baseline / 6 / 12 / 18 months	50 (1)	50 (1)	50 (1)	n/a
Biological Criteria					
Postoperative sensitivity	Baseline / 6 / 12 / 18 months	50 (1)	50 (1)	50 (1)	n/a
Secondary caries	Baseline / 6 / 12 / 18 months	50 (1)	50 (1)	50 (1)	n/a
Periodontal response	Baseline / 6 / 12 / 18 months	50 (1)	50 (1)	50 (1)	n/a
Pulp vitality	Baseline / 6 / 12 / 18 months	50 (1)	50 (1)	50 (1)	n/a

## Discussion

In restorative dentistry, clinical success relies on mechanical strength, long-term aesthetic stability, biological compatibility, and ease of application. However, no existing restorative material fits all these criteria, perpetuating the quest for a “ideal material.” Recent trends have moved toward bioactive systems that are meant to work with dental tissues, improve remineralization, and make clinical procedures easier. Glass-hybrid and alkasite materials are examples of this new generation of ways to fix issues.

The null hypothesis of the present study, which stated that there would be no significant difference in the 18-month clinical performance among alkasite, glass-hybrid, and resin composite materials, was not rejected as all three materials exhibited similar aesthetic, functional, and biological results, with no statistically significant differences among groups at any recall period.

Surface gloss, surface texture, and color stability constitute essential esthetic parameters that significantly influence patient satisfaction as well as the long-term clinical performance of posterior restorations. In the current study, while minor fluctuations in gloss/luster and color match were noted in the glass-hybrid and alkasite groups over time, these alterations were not statistically significant and remained within clinically acceptable parameters. Still, the slow rise in score-2 observations is worth noting because it could be a sign of early esthetic deterioration that will become more obvious over time. These findings are in accordance with previous clinical and laboratory evidence indicating that glass-hybrid materials demonstrate a higher tendency toward early discoloration and inferior color match compared with composite resin restorations, particularly in demanding intraoral environments or when exposed to chromogenic substances [22–24]. This behavior is closely linked to their underlying acid–base hybrid chemistry. Glass-hybrid materials rely on an ion-leachable fluoroaluminosilicate glass and a water-rich polyalkenoate matrix in which acid–base reactions, ion exchange, and material maturation continue over time [25, 26]. The hydrophilic polyacrylate network, together with the presence of loosely bound water, increases water sorption and facilitates the diffusion of pigments and dissolved ions into the surface and subsurface layers [26–29]. Moreover, the relatively lower degree of cross-linking and the susceptibility of the matrix to hydrolytic degradation and surface roughening may generate micro-porosities that further promote stain adsorption [26]. However, composite resin restorations consistently demonstrate the best color stability, followed by glass-hybrids being more prone to color changes due to their hydrophilic matrix and susceptibility to water sorption and staining [22, 30–32].

Alkasite restorations, on the other hand, showed better surface gloss and roughness outcomes compared with the glass-hybrid group, although not to the same extent as composite resin restorations in the present study. This finding corroborates existing literature demonstrating that alkasite exceeds glass-hybrid restorations in stain resistance and the maintenance of aesthetic qualities, particularly during the first year post-application [22, 33, 34]. The Alkasite’s hydrophobic UDMA-based resin matrix and tightly cross-linked filler network are what make these benefits possible. They help keep the surface glossy and smooth over time, and they make it less likely to absorb water and get stained. They also make the surface less likely to break down and get damaged. This is what makes it more color-stable than glass-hybrid materials in both clinical and laboratory settings [22, 33–35]. When we compare alkasite materials to composite resins, though, they tend to change color more, especially when they come into contact with coffee, children’s medicines, or acidic drinks [33, 35–37]. Composite resins consistently demonstrate better color stability than these other materials [22, 35, 37, 38].

All of the restorative materials in this study showed great functional stability over the 18-month follow-up period. This encompassed retention, marginal adaptation, wear resistance, preservation of proximal contact, and radiographic integrity. The consistent functional success across all groups indicates that, during this period, the intrinsic mechanical and interfacial properties of the materials were sufficient to withstand occlusal loading and intraoral stresses typical of Class II restorations. This finding is consistent with numerous randomized controlled trials and systematic reviews that demonstrate glass-hybrid, alkasite, and composite resin restorations consistently achieve retention rates exceeding 90% in Class II cavities over 18–24 months, thereby meeting or exceeding the performance standards established by the American Dental Association for definitive restorative materials. A recent multicenter study spanning five years revealed survival rates of 94.5% for glass-hybrid resin composite and 94.4% for nano-hybrid resin composite, with retention rates exceeding 93% for both materials at two years [39, 40]. At 12 months, alkasite-based restorations had the same survival rates as resin composites, and there were no major differences in retention or marginal adaptation [18]. Systematic reviews and meta-analyses validate that high-viscosity glass ionomer and composite resin restorations exhibit similar clinical efficacy in posterior teeth [41, 42]. During this period, no notable disparities in survival or success rates have been recorded between glass-hybrid and composite resin groups, a result corroborated by the current study. The favorable functional behavior of glass-hybrid systems is largely attributed to their optimized reactive glass network, improved flexural and cohesive strength thanks to highly reactive, finely sized glass particles compared to conventional glass ionomers, and chemical adhesion to tooth structure, combined with the absence of polymerization shrinkage associated with acid–base hardening, which minimizes interfacial stress and supports stable marginal adaptation [12, 43, 44].

Similarly, alkasite restorations have demonstrated functional outcomes comparable to composite resins in clinical trials, a performance that may be explained by their self- or dual curing mechanism ensuring a more uniform polymerization throughout the restoration, reduced polymerization stress due to the unique filler system and polymerization modulators, and balanced elastic modulus that is designed to be intermediate—stiff enough for load-bearing but flexible enough to absorb occlusal forces, promoting favorable stress distribution and interfacial stability under chewing loads, which together support stress distribution and interfacial stability under occlusal loading [45–47].

No secondary caries, postoperative sensitivity, or periodontal adverse reactions were observed for any of the materials during the 18-month follow-up in consistent with previous studies [39, 48]. The biological stability observed in this study may be explained by the fluoride release and buffering capacity of glass-hybrid and alkasite materials, their ability to chemically bond to surrounding tooth structures, and their capacity to reduce cariogenic activity at restoration margins [18, 49].

A major strength of the current clinical trial is that all restorative procedures are done in the same way. The use of strict rubber dam isolation and a single, highly trained operator made sure that the moisture was controlled as well as possible and that the technique was as consistent as possible, in addition to the materials’ natural properties. Strict control of the clinical setting strengthened the internal validity of the present study and likely contributed to the consistent functional outcomes observed across all restorative groups. The findings indicate that bioactive restorative materials, specifically glass-hybrid and alkasite systems, exhibit functional performance close to traditional composite resins in the short to medium term within this regulated context. By reducing the variability linked to operator technique and environmental influences, the observed results can be more reliably ascribed to the materials themselves rather than to external confounding variables.

Despite these favorable findings, several limitations should be considered when interpreting the results. An 18-month follow-up period provides insight into short-term clinical behavior; however, it may not be sufficient to fully reflect long-term material performance or degradation, particularly in relation to esthetic parameters such as color stability and surface integrity. In addition, the single-center, single-operator design, while supporting methodological consistency, may reduce the generalizability of the findings to everyday clinical practice involving operators with differing levels of experience and varying clinical conditions. Although the sample size was appropriate for identifying differences in functional outcomes, smaller yet clinically meaningful variations in esthetic durability may have remained undetected. Furthermore, the absence of patient-reported outcome measures and qualitative assessment of postoperative sensitivity limits interpretation from a patient-centered perspective.

Another limitation of the present study is the lack of quantitative standardization of cavity dimensions. While extremely small and excessively large cavities were excluded, some variation in cavity size and configuration among restorations was inevitable and may have influenced stress distribution and clinical outcomes. On the other hand, this variability mirrors routine clinical practice, where cavity morphology is dictated by lesion extent rather than predefined dimensions, which may actually increase the external validity of our results.

A significant advantage of this study is the direct comparison of alkasite, glass-hybrid, and composite resin materials under uniform oral conditions. By testing all three restorative materials on the same patient, we were able to reduce biological variability between individuals, which made it easier to judge their clinical performance without having to worry about differences between patients.

The study’s prospective and randomized design, the use of standardized clinical protocols, and the participation of a single operator enhanced the internal validity of the findings by minimizing variability associated with the operator and technique. Furthermore, the 18-month follow-up period facilitated the assessment of restoration behavior beyond the immediate postoperative phase, yielding insights into medium-term clinical performance. This study contributes clinically pertinent data to the limited literature on the comparative performance of alkasite, glass-hybrid, and composite resin materials under uniform clinical conditions. Although the present study provides clinically relevant outcomes over an 18-month period, longer follow-up is required to confirm long-term restorative performance. Therefore, the findings should be interpreted within the limits of the observation period.

## Conclusions

During the 18‑month observation period, glass-hybrid, alkasite, and resin composite restorations demonstrated comparable clinical performance in Class II cavities. All restorative materials exhibited clinically acceptable esthetic properties; however, resin composite showed the greatest color stability over time, followed by alkasite and glass-hybrid materials.

In terms of functional performance, all restorative materials were fully successful with respect to retention, marginal adaptation, proximal contact, and resistance to wear. Moreover, no cases of secondary caries, postoperative sensitivity, or adverse periodontal findings were observed in any of the study groups throughout the 18‑month follow-up. These findings indicate that all tested materials provide satisfactory short-term clinical performance in Class II cavities.

## Supplementary Information

Below is the link to the electronic supplementary material.

Supplementary Material 1 (JPG 7.29 MB)

Supplementary Material 2 (JPG 7.51 MB)

Supplementary Material 3 (JPG 9.72 MB)

Supplementary Material 4 (JPG 9.77 MB)

Supplementary Material 5 (JPG 10.8 MB)

Supplementary Material 6 (JPG 13.7 MB)

Supplementary Material 7 (JPEG 66.4 KB)

## Data Availability

The datasets generated and/or analyzed during the current study are available from the corresponding author on reasonable request.

## Declarations

### Clınıcal relevance

Tested bioactive restorative materials can be regarded as dependable substitutes for traditional composite resin in Class II restorations within regulated clinical settings.
