# Supplementary figures and images for: Clinical performance of alkasite and glass-hybrid restorations compared with resin composite in class II cavities: a randomized clinical trial
Source: Clin Oral Investig. 2026 May 2;30(5):211. doi: 10.1007/s00784-026-06869-w (PMC13134977; doi:10.1007/s00784-026-06869-w)

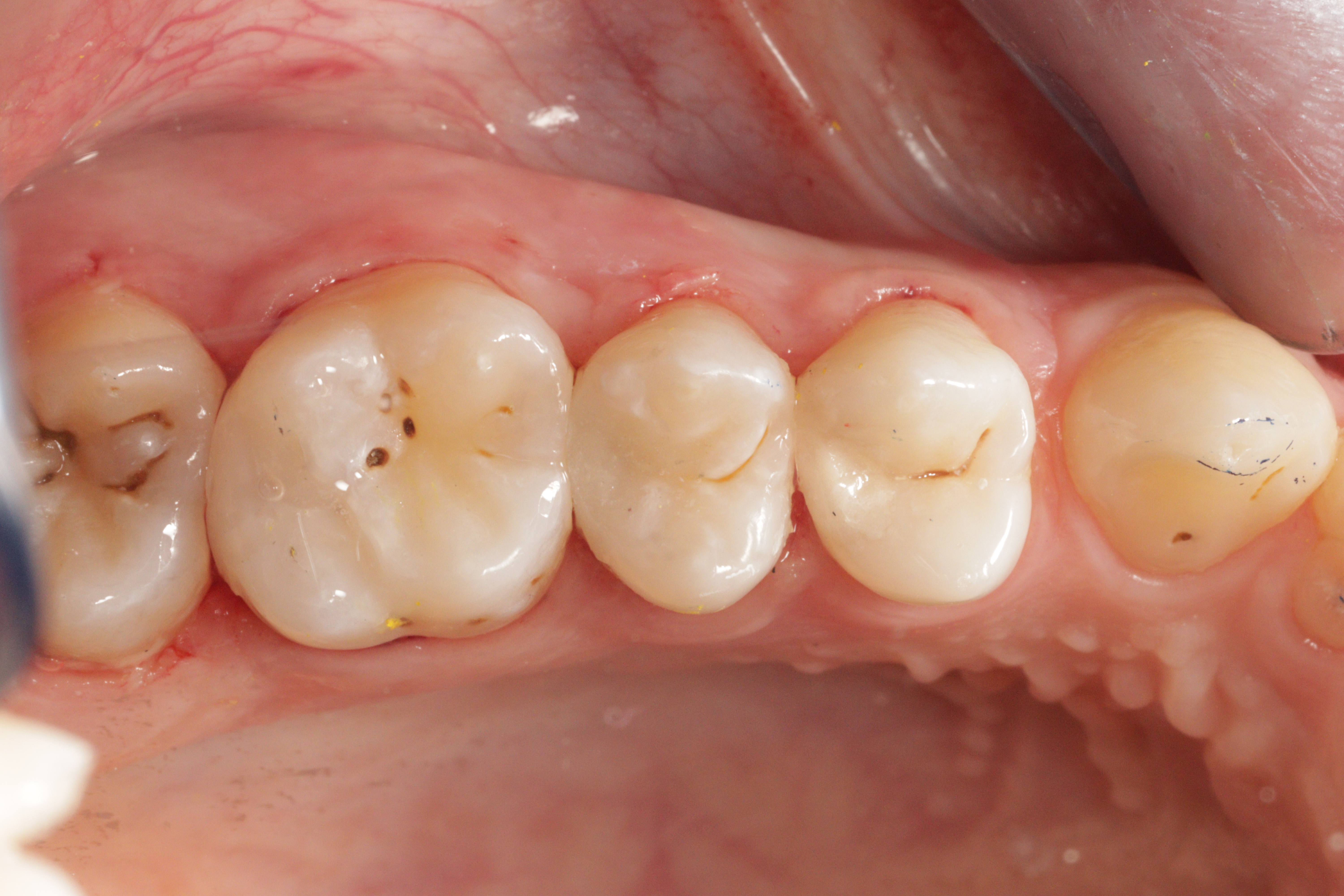

Supplement: Supplementary file 1 — Supplementary Material 1 (JPG 7.29 MB) [file 784_2026_6869_MOESM1_ESM.jpg]

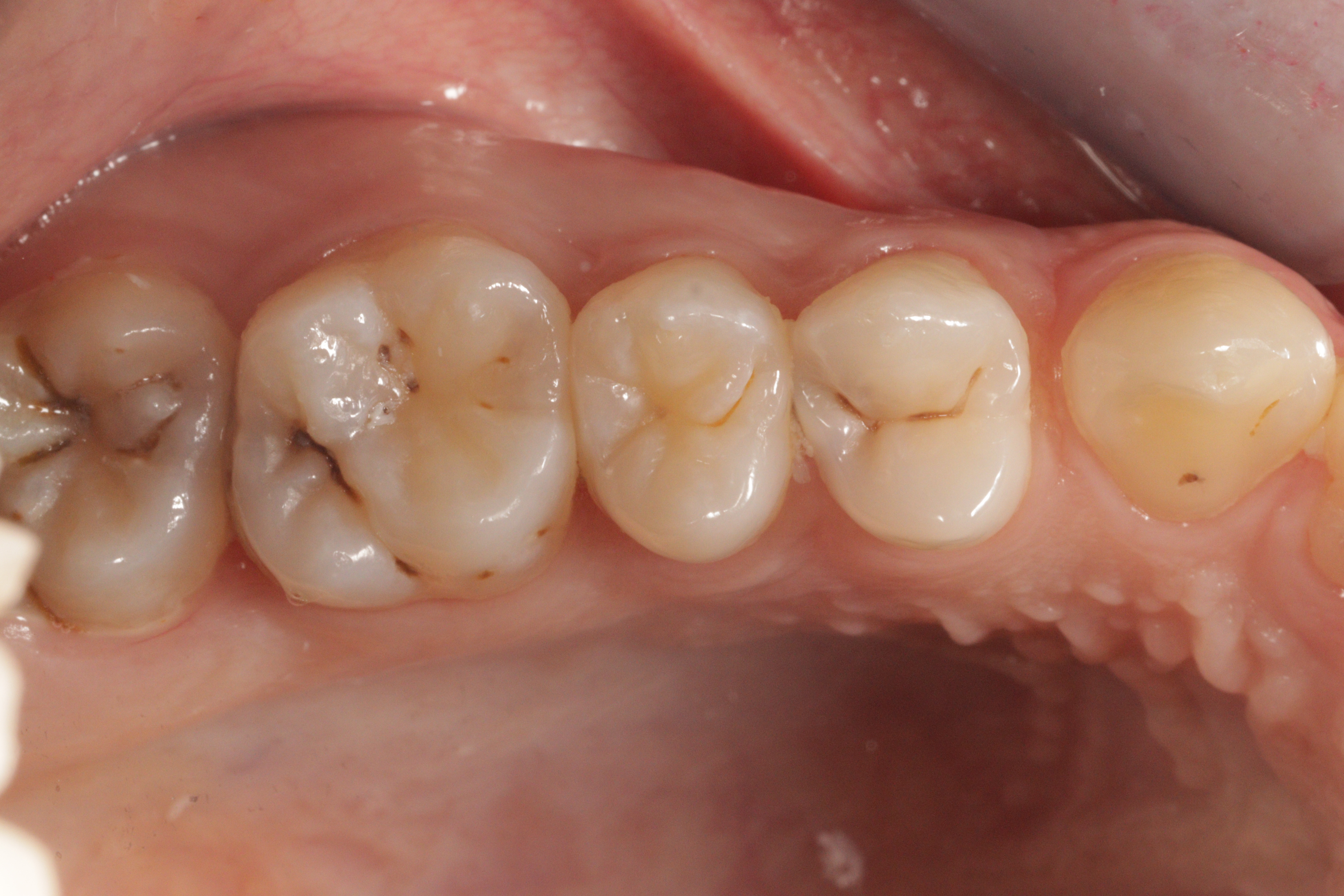

Supplement: Supplementary file 2 — Supplementary Material 2 (JPG 7.51 MB) [file 784_2026_6869_MOESM2_ESM.jpg]

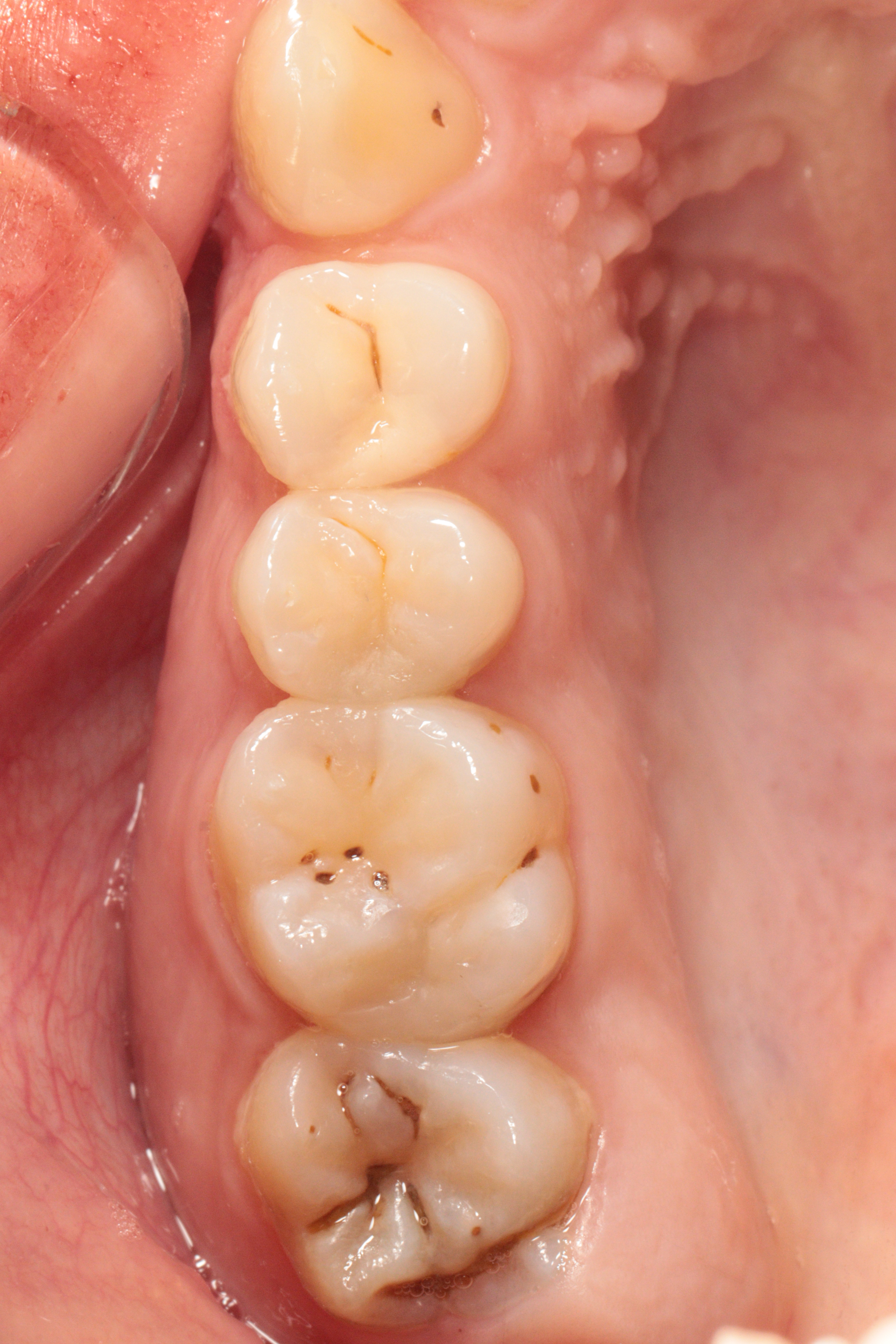

Supplement: Supplementary file 3 — Supplementary Material 3 (JPG 9.72 MB) [file 784_2026_6869_MOESM3_ESM.jpg]

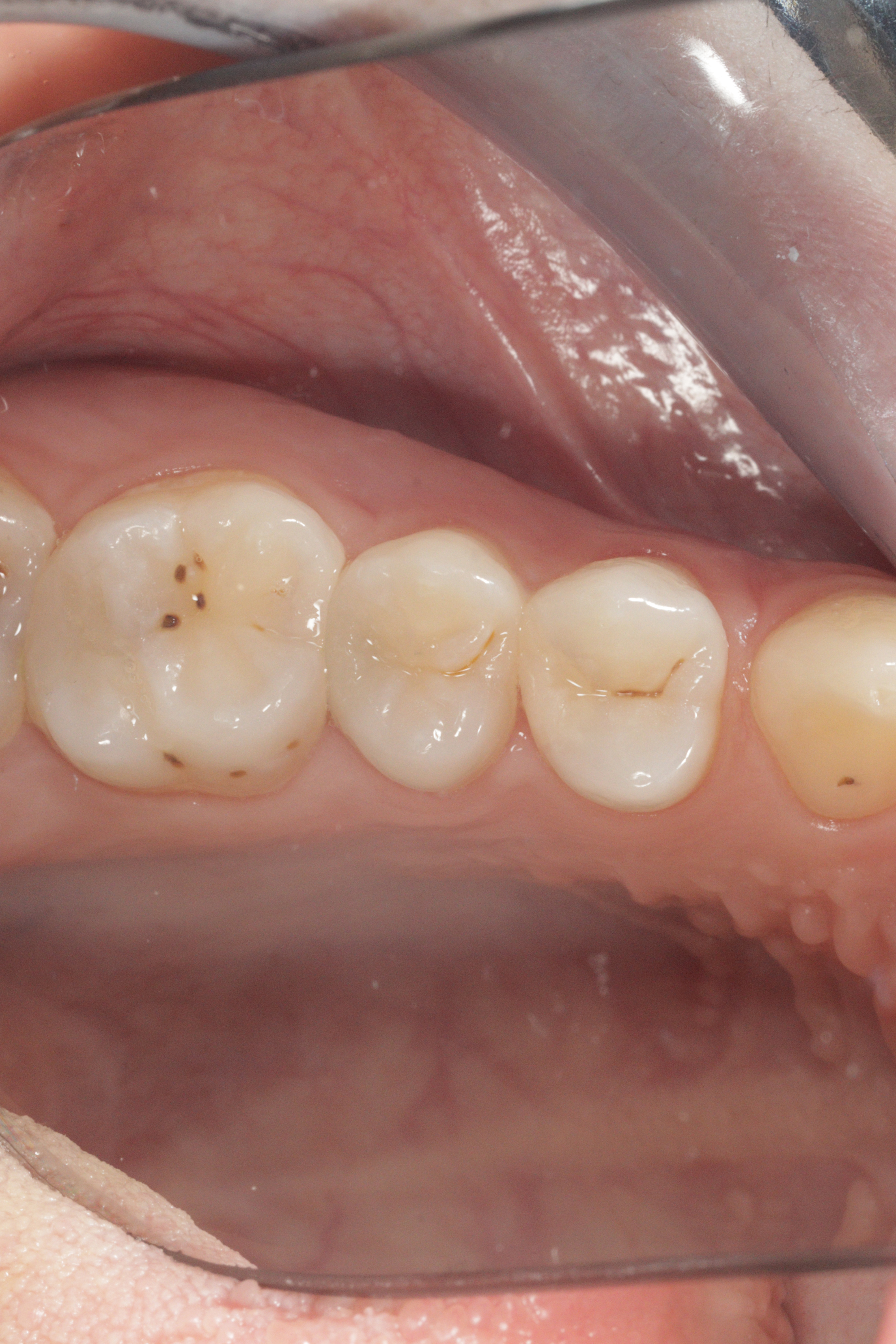

Supplement: Supplementary file 4 — Supplementary Material 4 (JPG 9.77 MB) [file 784_2026_6869_MOESM4_ESM.jpg]

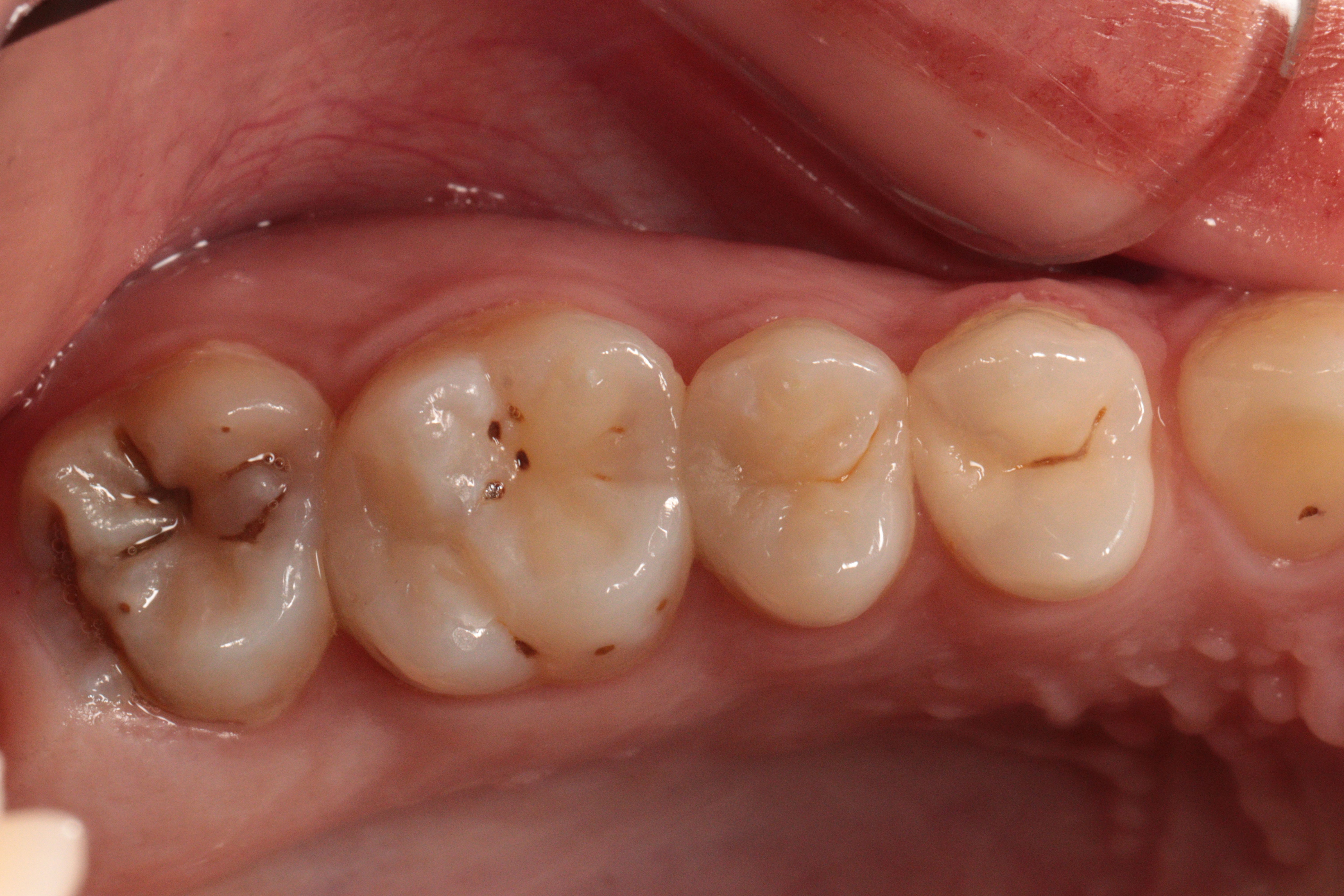

Supplement: Supplementary file 5 — Supplementary Material 5 (JPG 10.8 MB) [file 784_2026_6869_MOESM5_ESM.jpg]

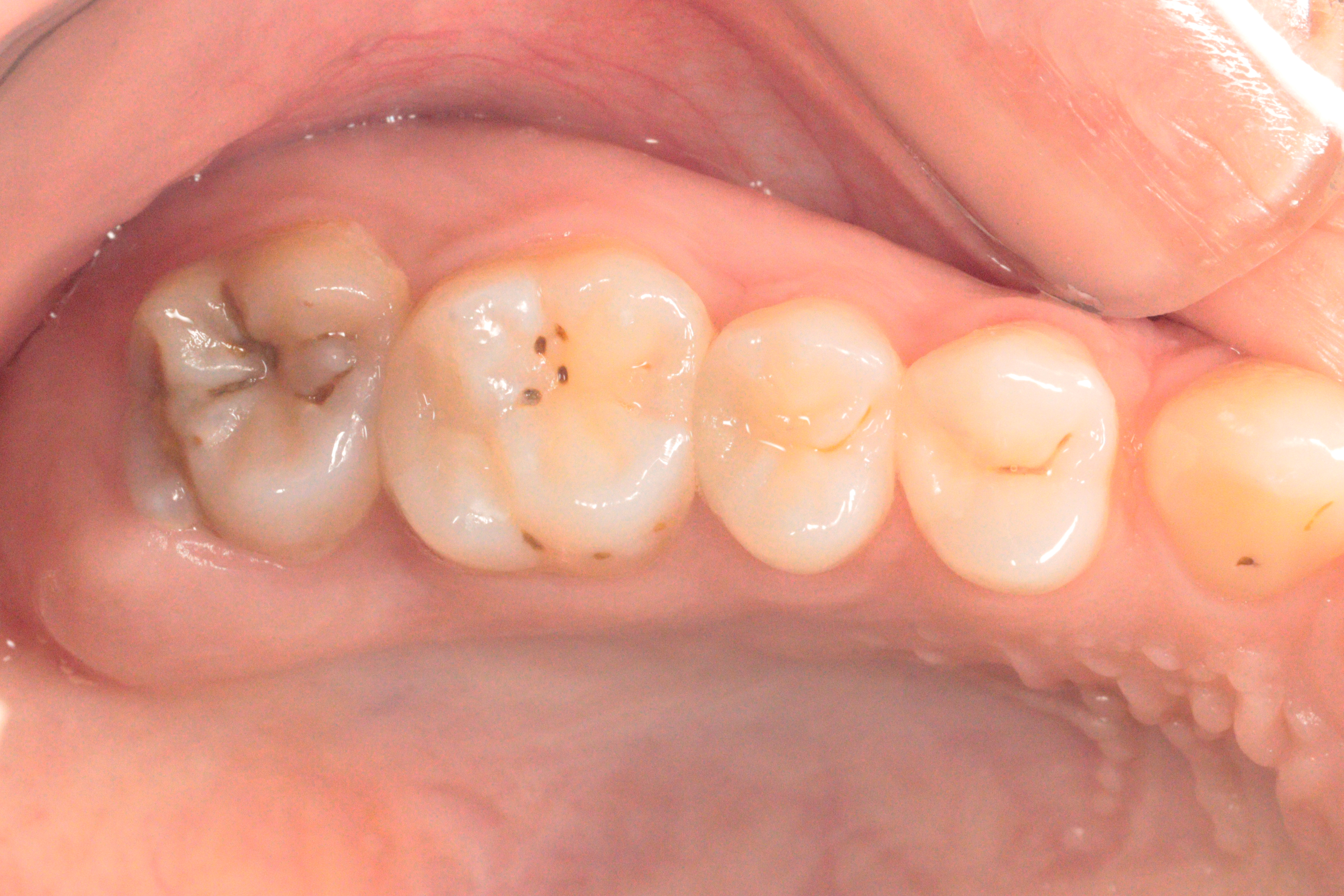

Supplement: Supplementary file 6 — Supplementary Material 6 (JPG 13.7 MB) [file 784_2026_6869_MOESM6_ESM.jpg]

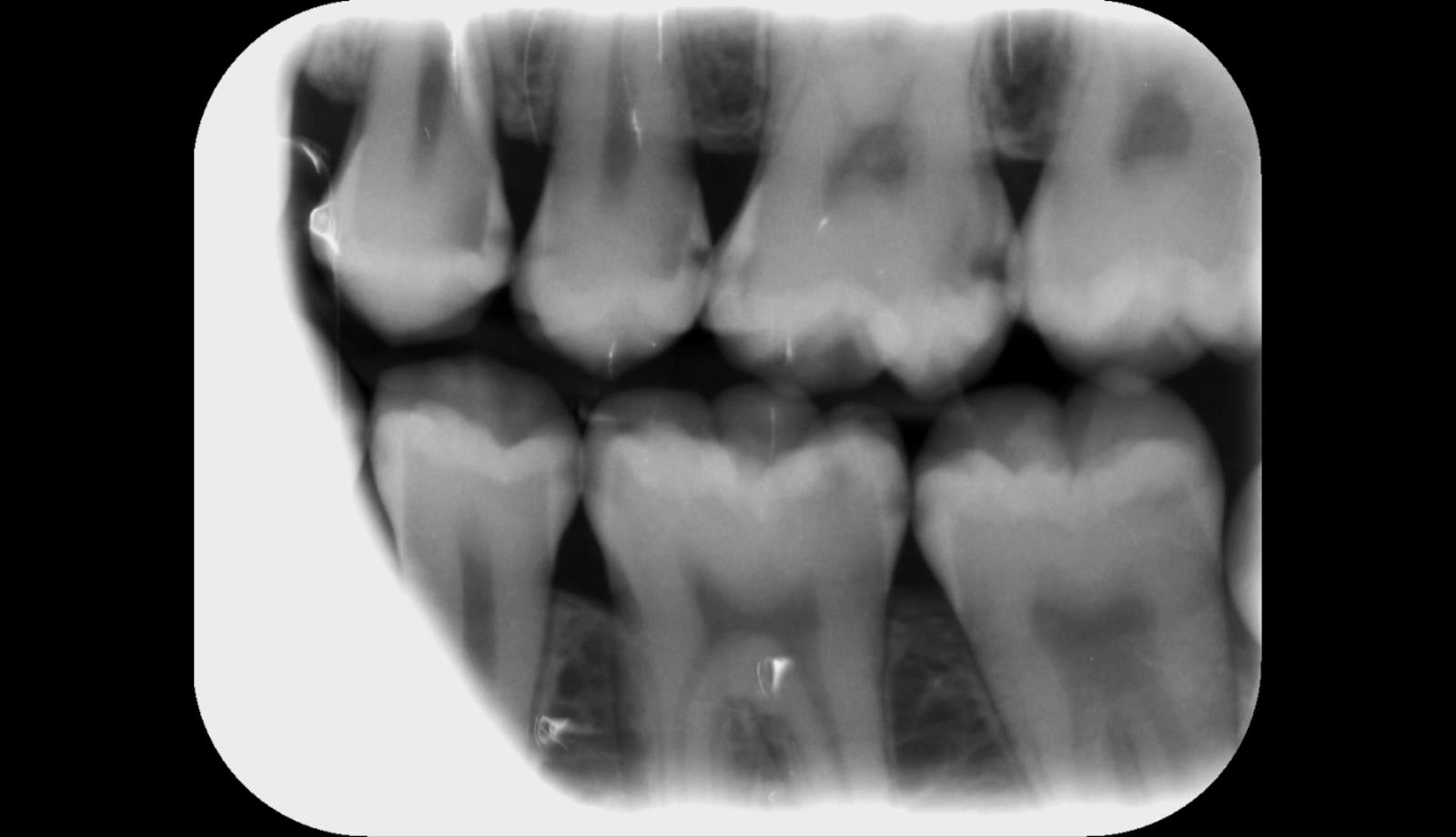

Supplement: Supplementary file 7 — Supplementary Material 7 (JPEG 66.4 KB) [file 784_2026_6869_MOESM7_ESM.jpeg]
